# Diagnosing Cellulitis of the Penis with Point-of-Care Ultrasonography in a Resource-Limited Setting

**DOI:** 10.1155/2023/1626736

**Published:** 2023-07-15

**Authors:** Yonathan Aliye Asfaw, Ayush Anand, Helen Huang, Muhammad Taimur, Sujan Poudel, Rajeswar Kumar, Mhmod Kadom, Sangam Shah, Gavrilo Lazovic, Ivan Rodriguez

**Affiliations:** ^1^Internal Medicine, Addis Hiwot Hospital, Addis Ababa, Ethiopia; ^2^Department of Research & Academic Affairs, Larkin Community Hospital, South Miami, USA; ^3^B. P. Koirala Institute of Health Sciences, Dharan, Nepal; ^4^University of Medicine and Health Sciences, Royal College of Surgeons in Ireland, Dublin, Ireland; ^5^Dow Medical College, Dow University of Health Sciences, Karachi, Pakistan; ^6^National medical College and Teaching Hospital, Birgunj, Nepal; ^7^Rajah Muthiah Medical College, Chidambaram, India; ^8^Tribhuvan University, Institute of Medicine, Maharajgunj 44600, Nepal; ^9^Department of Emergency, Larkin Community Hospital Palm Springs Campus, Hialeah, Florida, USA; ^10^Department of Family Medicine, Larkin Community Hospital South Miami Campus, Hialeah, Florida, USA

## Abstract

Cellulitis is a potentially serious bacterial skin infection. Penile cellulitis refers to the inflammation of the penile shaft and commonly occurs in uncircumcised, sexually active young adults. We reported the case of a 25-year-old heterosexual circumcised male patient with a two-day history of swelling and pain over the penile shaft. Local examination revealed a diffusely swollen penile shaft, erythematous, warm to the touch, and tender. The penile discharge culture was suggestive of a Streptococcus species infection. Ultrasonography of the penis showed increased echogenicity of the left side of the penile shaft soft tissue with a markedly increased Doppler signal, indicating cellulitis. Based on these findings, the patient was diagnosed with cellulitis of the penis and managed with broad-spectrum antibiotics. Though history and clinical examination are sufficient to diagnose penile cellulitis, our case highlighted that ultrasound could also support the diagnosis of penile cellulitis and help rule out differentials.

## 1. Introduction

Cellulitis is a potentially serious bacterial skin infection affecting the deeper dermis and subcutaneous connective tissue [1, 2]. It accounts for an incidence rate of 200 cases per 100,000 patient-years [2]. Arms, feet, and legs are not uncommon sites for infection [1, 2]. It can also develop around the eyes, mouth, anus, and rarely to the penis [1, 2]. Penile cellulitis refers to the inflammation of the penile shaft [1]. Although it most commonly occurs in uncircumcised, sexually active young adults, cases involving other age groups have been reported [3–5]. Signs and symptoms overlap with sexually transmitted infections (STIs); therefore, each case warrants a high index of clinical suspicion to rule out STIs [4]. In most cases, microbiological cultures are done to investigate the causative pathogen [3, 4, 6, 7]. However, conducting thorough laboratory investigations to isolate specific strains is not feasible in a low-income community setting [8]. A targeted approach with the help of point-of-care ultrasound can help yield improved diagnostic outcomes [9]. Our case highlights the unique challenge of diagnosing penile cellulitis and differentiating it from similarly presenting conditions in a resource-limited setting with the help of point-of-care ultrasound.

## 2. Case Report

A 25-year-old heterosexual circumcised male patient presented to the emergency department with a two-day history of swelling of the penile shaft. He noticed a gradually worsening penile shaft swelling with associated mild pain that started two days ago. The patient did not have signs/symptoms of dysuria, urgency, frequency, and hesitancy of urine flow. However, he had a positive history of unprotected sexual intercourse two weeks back with a female partner. He reported always being in a monogamous relationship with his female partner, who did not report any active vaginal discharge or vulvar lesions that may suggest provisional findings of any sexually transmitted infection.

On general physical examination, the patient seemed well, with no evidence of systemic signs of infection like fever, chills, altered mental status, tachycardia, or hypertension. On local examination, there was a diffusely swollen penile shaft, erythematous, warm to the touch, and tender. There were no ulcerations on the penile shaft and associated lymphadenopathy. Scant, nonbloody, purulent discharge from the external urethral orifice and on the exterior of the penile shaft was noted. The site of infection was evident.

**Figure 1:**
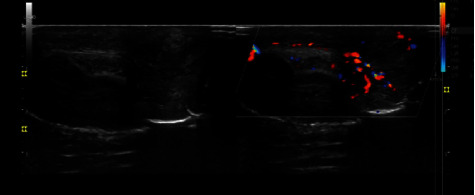
Ultrasound of the penis showing increased color doppler signal on the left side of the penile shaft compared to the right side of the shaft.

**Figure 2:**
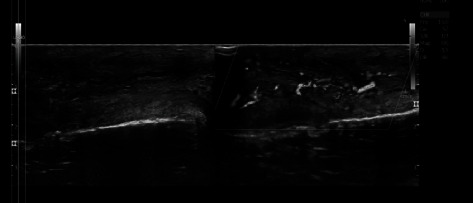
Ultrasound of the penis showing increased echogenicity on the left side of the penile shaft compared to the right side of the shaft.

The patient's urinalysis showed 20-30 WBCs/HPF, and his complete blood counts were normal. venereal disease research laboratory tests (VDRL), rapid plasma reagin (RPR), wet mount, Human Immunodeficiency Virus (HIV), and rapid antibody tests were negative. The penile discharge culture revealed gram-positive cocci in chains, indicating Streptococcus species infection. Ultrasound of the penis (Figures 1 and 2) showed increased echogenicity of the left side of the penile shaft soft tissue with markedly increased doppler signal, indicating cellulitis. Based on the clinical, laboratory, and ultrasonography findings, the patient was diagnosed with penile cellulitis. The patient was treated with 1 gm per day of oral amoxicillin-clavulanic acid for a week. On follow-up after one week, there was a significant improvement in penile swelling and associated symptoms. He did not report any complications or worsening conditions during the outpatient visit, including erectile dysfunction, tenderness, ulceration, or discharge from the penis.

## 3. Discussion

Penile cellulitis is an emergency and rare clinical entity that usually affects young, uncircumcised, and sexually active men [3]. However, it can affect all age groups, including infants [4, 5]. Therefore, a diagnostic approach with high clinical suspicion was warranted to rule out generalized and local causes of penile swelling and discharge, including STIs and general inflammatory conditions caused by non-STIs [4]. It is crucial to take a detailed sexual history relevant to STIs, including unprotected sexual intercourse, multiple sexual partners, oral sex, history of STIs, intravenous drug abuse, and men who have sex with men [8]. In addition, a history of trauma and allergies should be inquired about to rule out general inflammatory skin conditions leading to penile swelling [2]. Commonly, the spectrum of clinical presentation of penile cellulitis overlapped with sexually transmitted infections and was the point of contention in our patient [3–5, 10]. Usually, the patient diagnosed with an STI presents with penile swelling, localized pain, and either purulent or watery discharge [3, 4, 7, 10]. As a result, specific investigations for STIs should be carried out to rule out sexually transmitted diseases [8]. In our case, specific investigations such as VDRL and RR were carried out with a complete history of sexual encounters, which aided in the differential diagnosis of our patient. There may be associated urinary symptoms such as pain during micturition, urgency, and hesitancy with STIs, and must be ruled out from urinary tract infections upon microbiology culture results and urinalysis [7].

In our case, Streptococci were reported as the causative pathogen of penile cellulitis and remain the most common organism isolated in skin cellulitis [3]. In the literature, there are only four reported cases of penile cellulitis caused by streptococci organisms [3, 4, 6, 7]. These case reports relied on clinical evaluation and microbiology cultures of the discharge for confirmation of penile cellulitis [3, 4, 6, 7]. Though our culture also revealed streptococci species as the causative pathogen, extensive and elaborate laboratory investigations required to isolate the strain are often not possible in resource-limited settings because they are not accessible and more expensive ^8^. This was applicable in our case, as the patient did not opt for a further microbiology investigation due to healthcare expenses. Under these circumstances, it can be difficult to definitively diagnose penile cellulitis and provide the best course of treatment without isolating the exact stain of microbiology cultures [4, 8]. However, a point-of-care ultrasound (POCUS) of the penis in our case helped visualize fluid collection and the echogenicity of penile soft tissue, along with clinical findings, further supporting the definitive diagnosis of cellulitis [8, 10–12]. POCUS was indicated in our case as a supporting modality to support a diagnosis and significantly avoid additional costs associated with extensive laboratory investigations for our patient [10–12]. To our best knowledge, this was one of the first cases reported in the literature to utilize POCUS as a supportive imaging modality in penile cellulitis. POCUS has been a well-sought diagnostic tool for other forms of skin cellulitis and draws more attention to the primary care physician in the present medical era. A prospective observational study by Knaysi et al. conducted in 2020 at a tertiary medical center revealed that POCUS increases the ability to rule in the diagnosis and directs the effective transition to the management in patients with skin and soft tissue infections (SSTI) [13]. On the other hand, a systematic review of 644 articles by Becker et al. conducted in 2016 revealed that portable ultrasound devices are being successfully used to triage, diagnose, and treat patients with a wide variety of complaints in the low- to middle-income countries [14]. These studies highly advocate for the wise use of POCUS for initial diagnosis in a resource-limited setting. Moreover, POCUS can provide a prompt assessment to initiate treatment right away, a critical aspect of care that impacts these patients' overall prognosis [4]. Though POCUS can help in confirming the diagnosis, it is neither essential nor can it be a substitute to proper history and examination, which are sufficient to reach a diagnosis of cellulitis. Also, etiological diagnosis can only be made through microbiological tests, based on which indicated antibiotics can be used.

Typically, penicillin is preferred as outpatient therapy for streptococcal infection in the absence of features of systemic signs of infections. [15] However, empirical therapy with broad-spectrum antibiotics such as amoxicillin-clavulanate can help prevent local and systemic complications. [4, 15] In our case, amoxicillin-clavulanate was used, which improved the condition, and the patient returned to his baseline health.

## 4. Conclusion

Our case highlights the diagnosis of penile cellulitis caused by streptococcus in a heterosexual adult male patient. The diagnosis should be made based on an exclusion of differentials and a high index of clinical suspicion, as signs and symptoms of penile cellulitis can overlap with an STI or inflammatory skin conditions. In low-resource settings, point-of-care ultrasonography can support the diagnosis of penile cellulitis along with cultures and should be routinely used to avoid the additional costs of advanced-technology investigations for patients in low-income communities.

## Data Availability

This manuscript has all the data relevant to this case report included.
